# **Problematization methodology’s impact on nursing and medical students’ knowledge about hypodermoclysis: quasi-experimental study** *

**DOI:** 10.1590/1518-8345.7006.4131

**Published:** 2024-03-15

**Authors:** Juliana de Souza Lima Coutinho, Érica Toledo de Mendonça, Luciene Muniz Braga, Patrícia de Oliveira Salgado, Flavia Falci Ercole, Luana Vieira Toledo

**Affiliations:** ^1^ Universidade Federal de Minas Gerais, Escola de Enfermagem, Belo Horizonte, MG, Brazil; ^2^ Universidade Federal de Viçosa, Viçosa, MG, Brazil

**Keywords:** Nursing Students, Medical Students, Hypodermoclysis, Educational Technology, Knowledge, Teaching, Estudiantes de Enfermería, Estudiantes de Medicina, Hipodermoclisis, Tecnología Educacional, Conocimiento, Enseñanza, Estudantes de Enfermagem, Estudantes de Medicina, Hipodermóclise, Tecnologia Educacional, Conhecimento, Ensino

## Abstract

**Objective:**

to analyze the problematization methodology impact on the knowledge of nursing and medical students about hypodermoclysis.

**Method:**

quasi-experimental study conducted with 22 undergraduate nursing and medical students from a public Brazilian higher education institution. The students participated in the educational intervention using the problematization methodology based on the Arch of Maguerez. A previously validated questionnaire was used to determin’ the students’ knowledge level about hypodermoclysis. This instrument was applied before and after the educational intervention. The results were compared by McNemar’s test and Student’s t test for paired samples.

**Results:**

when comparing the correct answers before and after the intervention, there was a significant increase in 75% of the questions (p<0.05), including theoretical and practical aspects of hypodermoclysis. The mean score on students’ self-assessment of the ability to explain (0.9 versus 5.9 points) and perform hypodermoclysis (1.9 versus 5.0) was significantly higher after applying the problematization methodology (p<0.001).

**Conclusion:**

the problematization methodology had a positive impact on the students’ knowledge about hypodermoclysis. The number of correct answers after the educational intervention was higher than the initial assessment. The problematization methodology can be incorporated into the teaching-learning process of nursing and medical students for teaching procedures such as hypodermoclysis.

## Resumen

**Objetivo:**

analizar el impacto de la metodología de problematización en el conocimiento de estudiantes de enfermería y medicina sobre hipodermoclisis.

**Método:**

estudio cuasiexperimental realizado con 22 estudiantes de enfermería y medicina de una institución pública de educación superior brasileña. Los estudiantes participaron de la intervención educativa utilizando la metodología de problematización basada en el Arco de Maguerez. Se utilizó un cuestionario previamente validado para determinar el nivel de conocimiento de los estudiantes sobre la hipodermoclisis. Este instrumento se aplicó antes y después de la intervención educativa. Los resultados se compararon mediante la prueba de McNemar y la prueba t de Student para muestras pareadas.

**Resultados:**

al comparar las respuestas correctas antes y después de la intervención, se observó un aumento significativo en el 75% de las preguntas (p<0,05), que incluye aspectos teóricos y prácticos de la hipodermoclisis. El puntaje promedio en la autoevaluación de los estudiantes con respecto a su capacidad para explicar (0,9 versus 5,9 puntos) y realizar hipodermoclisis (1,9 versus 5,0) fue significativamente mayor después de aplicar la metodología de problematización (p<0,001).

**Conclusión:**

la metodología de problematización tuvo impacto positivo en el conocimiento de los estudiantes sobre la hipodermoclisis. El número de respuestas correctas luego de la intervención educativa fue mayor que en la evaluación inicial. La metodología de problematización puede incorporarse al proceso de enseñanza-aprendizaje de estudiantes de enfermería y medicina para enseñar procedimientos como la hipodermoclisis.

## Resumo

**Objetivo:**

analisar o impacto da metodologia de problematização no conhecimento de estudantes de enfermagem e medicina sobre hipodermóclise.

**Método:**

estudo quase-experimental realizado com 22 estudantes de graduação em enfermagem e medicina de uma instituição pública de ensino superior brasileira. Os alunos participaram da intervenção educacional usando a metodologia de problematização baseada no Arco de Maguerez. Um questionário previamente validado foi usado para determinar o nível de conhecimento dos alunos sobre hipodermóclise. Esse instrumento foi aplicado antes e depois da intervenção educacional. Os resultados foram comparados pelo teste de *McNemar* e pelo teste t de *Student* para amostras pareadas.

**Resultados:**

ao comparar as respostas corretas antes e depois da intervenção, houve um aumento significativo em 75% das questões (p<0,05), incluindo aspectos teóricos e práticos da hipodermóclise. A pontuação média na autoavaliação dos alunos quanto à capacidade de explicar (0,9 versus 5,9 pontos) e realizar a hipodermóclise (1,9 versus 5,0) foi significativamente maior após a aplicação da metodologia de problematização (p<0,001).

**Conclusão:**

a metodologia de problematização teve um impacto positivo no conhecimento dos alunos sobre hipodermóclise. O número de respostas corretas após a intervenção educacional foi maior do que na avaliação inicial. A metodologia de problematização pode ser incorporada ao processo de ensino-aprendizagem de estudantes de enfermagem e medicina para o ensino de procedimentos como a hipodermóclise.

**Highlights:**

**(1)** The problematization methodology had a positive impact on students’ knowledge.

**(2)** The number of correct answers after the educational intervention increased.

**(3)** Problematization can be incorporated into the teaching of nursing and medicine.

**(4)** The teaching-learning process through active methodologies should be encouraged.

**(5)** Problematization has the potential to develop cognitive and attitudinal skills.

## Introduction

Hypodermoclysis refers to a procedure/technique to access the subcutaneous route, enabling the medications and fluids administration. This procedure has been used in patients who need hydration and symptom control, in cases where there are impediments to oral or venous access ^(^ ^1^ ^-^ ^2^ ^)^. Older adults patients and/or patients under palliative care have been the target of this intervention due to clinical conditions such as dehydration or fragility of peripheral blood vessels, as it is safe and effective for these patients ^(^ ^2^ ^-^ ^3^ ^)^. 

Among the hypodermoclysis advantages are its low cost, low risk of complications and adverse events, and its easy insertion and maintenance ^(^ ^1^ ^,^ ^4^ ^-^5 ^)^. Another considerable advantage is that its use prevents the patient from being subjected to several punctures, as happens in the intravenous route ^(^ ^6^ ^)^. 

Despite its benefits, there is low adherence to the hypodermoclysis use, as well as little dissemination of its technique, especially in the academic environment ^(^ ^7^ ^)^, in addition to predominance of the use of the venous route over the subcutaneous route for medication administration, even in patients with indication for the subcutaneous route ^(^ ^7^ ^)^. These findings may be related to the lack of information among professionals, resulting in insecurity and, consequently, lower adherence to this medication administration route ^(^ ^5^ ^)^. 

In this context, the teaching of hypodermoclysis during professional training is necessary, not only regarding the aspects of performing the technique, but also the critical-reflective thinking related to its indications. For this, new teaching methodologies have been sought, capable of stimulating the development of critical thinking skills in students, as well as their decision-making capacity. Among the various existing teaching methodologies, problematization stands out, as it is considered capable of stimulating professional autonomy, independence in the search for knowledge, critical thinking, and decision-making ^(^ ^8^ ^-^ ^9^ ^)^. 

Problematization is a teaching methodology that consists of observing reality and proposing a solution to an observed problem. This methodology comprises five stages: reality observation, key points survey, theorization, solution hypothesis and application to reality ^(^ ^10^ ^)^. 

Given the researchers’ experience with this methodology, it is believed that it can contribute to the training of professionals who are agents of change in their work environments ^(^ ^9^ ^)^. In addition, studies addressed the successful use of this methodology in teaching administrative and managerial issues, in addition to technical procedures such as peripheral venous access puncture ^(^ ^9^ ^)^. 

Thus, in view of the low adherence of hypodermoclysis in health services, possibly associated with weaknesses in the professionals’ knowledge, added to the relevance of its applicability in this sociodemographic transition context, with consequent increase of patients who may benefit from this route of infusion of medications and fluids, it is relevant to develop studies that seek to equip future professionals for decision-making and performance of this important therapeutic measure. Considering that graduation is a crucial moment for the acquisition of theoretical and practical knowledge and that problematization has been used as a teaching methodology for different topics in health, there is a need for research to evaluate its contributions on the teaching of hypodermoclysis.

In this context, this study is proposed to analyze the impact of the problematization methodology on the knowledge of nursing and medical students about hypodermoclysis.

## Method

### Study type

This is a quasi-experimental study, of the before-and-after type, in which all participants were exposed to the educational intervention and had the outcomes assessed.

### Location

The study was conducted in a public institution of higher education located Brazil’s southeastern region. This institution offers undergraduate courses in nursing and medicine, which annually receives students approved in the unified selection process. At that university, content on the hypodermoclysis technique is included in the Nursing Skills and Laboratory applied to the clinic disciplines.

### Period

The study was carried out in February 2022.

### Population

The study population was composed of final-year nursing and medical students enrolled in the aforementioned institution (n = 73 students).

### Selection criteria

Those aged 18 years or older were included. It was adopted as exclusion criterion the previous participation in specific training on the subject of hypodermoclysis. However, no student was excluded.

### Participants

All students were informed about the study objectives and invited to participate by signing the informed consent form. In the end, the non-probabilistic, intentional sample was composed of 22 students who attended the university on the day of the educational intervention.

### Instruments used for data collection and study variables

Before starting the educational intervention, students were allocated to school desks lined up in a classroom and answered the identification form containing gender, age, marital status, and academic background. Then, they answered the pre-test on their knowledge about hypodermoclysis, based on a previously validated instrument ^(^ ^11^ ^)^. It should be noted that, while filling out the forms, the students remained two meters away from each other, so it was not possible to check the answers provided by their colleagues. 

This instrument comprises four structured, multiple-choice questions about the theoretical aspects of hypodermoclysis: 1) What is hypodermoclysis? 2) Indications; 3) Absolute contraindications and 4) Relative contraindications. Regarding the practical aspects of hypodermoclysis, eight multiple-choice questions are presented: 1) Puncture sites; 2) Possible complications; 3) Professional responsible for prescribing; 4) Professional responsible for the puncture; 5) Device to be used for puncture; 6) Maximum time for the device to be needled; 7) Maximum time for the device to remain unstuck and 8) Maximum volume to be infused in 24 hours. In addition to the multiple-choice questions, the instrument presents two Likert-type questions referring to the self-assessment of the student’s knowledge and ability on the topic: 1) Do you consider that you have the technical ability to explain the procedure to other professionals (Likert-type scale from 0 to 10 points, with 0 not capable and 10 capable) and 2) You consider yourself capable of performing the technique (Likert scale from 0 to 10 points, 0 not capable and 10 capable).

### Data collection

The educational intervention was performed at the university itself, in the same room where the initial data collection instruments were filled out, and outside regular class hours. The students were randomly divided into three groups, with up to eight students in each group. Randomization was done by a draw, conducted by an external researcher. The three groups remained in the same room, but each one was allocated a separate place to start the application of the problematization methodology.

The Intervention was carried out following the five stages of the Arch of Maguerez: reality observation, key points survey, theorization, solution hypothesis and application to reality ^(^ ^12^ ^)^. 

Initially, in the reality observation stage, all students received a clinical case, constructed by the researchers, and validated by three specialists, and were instructed to perform a critical and reflective reading of the situation described. The case presented the contextualized history of a patient who had already experienced several hospitalizations both for personal problems and for her husband’s problems and, currently, was in palliative care, with uncontrolled symptoms, intense pain, and desire to remain at home. The clinical case’s central problem was the need for home treatment of symptoms by an alternative route to the oral and venous route, and also involved discussions about the cultural, psychological, affective, social, bioethical and financial aspects.

Then, based on the group discussion, the key points of the clinical case were listed, considering the multiple dimensions involved in the case. The researcher transcribed all the key points listed by each group onto a whiteboard. The key points were consolidated in a learning question, elaborated in agreement among all groups: What strategies can be used by the multiprofessional team in comprehensive care, ensuring comfort, autonomy and respecting the family context and subjectivity of the patient?

After the key points were identified and the learning question was elaborated, the third stage, called theorization stage, was carried out. Students were encouraged to search the scientific literature for information about the key points of the problem identified. For this, access to physical books and computers for internet access were made available. After the period for individual study, the groups met and made a knowledge’s synthesis. In each group, a reporter was selected to present the scientific evidence to the other students. The presentation was mediated by the researcher.

Afterwards, the groups listed hypothetical solutions to the problem-solving situation included in the learning question. The facilitator recorded all the suggestions made by each group on the whiteboard. It was not possible to apply it to reality *in loco* due to the impossibility of directing students to a real practical scenario during the educational activity. However, the hypotheses for solutions were presented by the groups, with details of their planning, so that they could be implemented by the students when they experienced this reality during their undergraduate internship or in their professional lives, as set out in Figure 1. 

**Figure 1:** - Operationalization of the stages of the quasi-experimental study. Viçosa, MG, Brazil, 2022

**Stages (Duration)**	**Goals**	**Performed activities**
Pre-test (30 minutes)	Assess students’ prior knowledge	- Application of the questionnaire on students’ knowledge about hypodermoclysis.
Reality observation (30 minutes)	Allow students to approach the topic through critical and reflective reading of the clinical case	- Critical and reflective reading of the situation reported in the clinical case of the patient in palliative care; - Clinical, critical and holistic observation of the situation described in the case; - Identification of the problem: need to treat symptoms using an alternative route to the oral and intravenous route.
Key points survey (60 minutes)	Reflect on the problems identified in the case and define the key points for the investigation	- Group discussion about the case; - Identification of the cases’ key points; - Elaboration of a learning question based on the cases’ key points.
Theorization (270 minutes)	Investigate the topic in scientific literature and compare previous knowledge and new information to solve the problem	- Contact with printed and digital scientific literature; - Synthesis of scientific evidence that responds to the problems identified in the clinical case; - Discussion on the topic between students and facilitator.
Solution hypotheses (60 minutes)	Develop suggestions to solve the clinical case problem	- Suggestion of solutions for the situation described in the clinical case.
Application to reality (60 minutes)	Promote the resolution of the problem presented in the clinical case	- Presentation of detailed planning of the solution hypotheses for the case, so that they can be reproduced in practice.
Post-test (30 minutes)	Assess students’ knowledge after the intervention	- Application of the questionnaire on students’ knowledge about hypodermoclysis.

### Data treatment and analysis

After the end of the intervention, the post-test was applied to measure the knowledge obtained from the intervention. The same previously validated instrument of the pre-test was used ^(^ ^11^ ^)^. 

Data were double-entered into Microsoft Excel and analyzed using the Statistical Program for the Social Sciences (SPSS version 22). A descriptive analysis of the students’ characterization data was performed. Categorical variables were expressed by absolute and relative frequencies. Quantitative variables were presented based on measures of central tendency and variability (mean and standard deviation or median and interquartile range), according to the normality of data distribution. The normality test used was the Kolmogorov-Smirnov.

To compare the knowledge of nursing and medical students about hypodermoclysis, the comparison of the question’s correct answers in the pre and post-test was performed using McNemar’s test. The judgment analysis of self-technical ability to explain the procedure to another student and to perform the procedure was performed from the comparison of the mean scores indicated by students in two moments: before and after the educational intervention, by paired Student’s t-test. It was adopted as significant p<0.05.

### Ethical aspects

The study was approved by the Research Ethics Committee of the proposing institution, opinion number 5.249.949 and the ethical aspects were respected.

## Results

Of the 22 students evaluated in this study, most were female (77.3%), single (86.4%) and enrolled in the undergraduate Nursing course (90.9%). The student’s mean age was 24.9 (±2.2) years.

In relation to the students’ knowledge about hypodermoclysis, it was found that of the 12 questions assessed, nine (75%) showed an increase of correct answers between pre and post-test (p<0.05). Only the questions referring to the concept of hypodermoclysis, possible complications and maximum volume infused in 24 hours showed no difference between the number of correct answers in the pre- and post-test, as shown in Table 1

**Table 1:** - Comparison of the number of correct answers of students to questions about hypodermoclysis before and after the educational intervention. Viçosa, MG, Brazil, 2022

**Variables**	**Pre-test n(%)**	**Post-test n(%)**	**p-value** *
*Theoretical aspects of hypodermoclysis*			
1. What is hypodermoclysis?	20 (90,9)	22 (100)	0,500
2. Indications	1 (4,5)	9 (40,9)	0,021
3. Absolute contraindications	-	6 (27,3)	0,031
4. Relative contraindications	-	6 (27,3)	0,031
*Practical aspects of hypodermoclysis*			
1. Puncture sites	2 (9,1)	15 (68,2)	<0,001
2. Possible complications	2 (9,1)	2 (9,1)	1,000
3. Responsible for the prescription	5 (22,7)	14 (63,6)	0,012
4. Person responsible for the puncture	4 (18,2)	14 (63,6)	0,004
5. Device used for puncture	7 (31,8)	18 (81,8)	0,001
6. Dwell time needled operated device	1 (4,5)	17 (77,3)	<0,001
7. Dwell time non-needle operated device	-	19 (86,4)	<0,001
8. Maximum volume to be infused in 24h	7 (31,8)	13 (59,1)	0,109

* McNemar test (p-value <0.05 - 95% significance)

In relation to the theoretical aspects of hypodermoclysis (concept, indications, and contraindications) it was found that after the intervention, there was an increase in the students’ correct answers. However, among the indications, only the impossibility of oral ingestion differed significantly between pre- and post-test (36.4% versus 95.5%; p<0.001). Regarding absolute contraindications, anasarca was considered by 81.8% of the students in the post-test but did not present significant difference in relation to the pre-test (59.1%; p= 0.180). Similarly, the relative contraindication related to the presence of areas of infection, inflammation, or skin lesion also did not differ statistically between the pre- and post-test (68.2% versus 81.8%; p= 0.375), as shown in Table 2. 

**Table 2:** - Frequency of student responses on theoretical aspects of hypodermoclysis before and after the educational intervention. Viçosa, MG, Brazil, 2022

**Theoretical aspects of hypodermoclysis**	**Pre-test n(%)**	**Post-test n(%)**	**p-value** *
**1. What is hypodermoclysis?**			
a) Intramuscular fluids and medications application’s route	1 (4,5)	-	1,000
b) Intravenous fluids and medications application’s route	1 (4,5)	-	1,000
c) Subcutaneous fluids and medications application’s route	20 (90,9)	22 (100)	0,500
**2. Indications (admits more than one correct alternative)**			
a) Inability to be ingested orally	8 (36,4)	21 (95,5)	<0,001
b) Impossibility of venous access	17 (77,3)	21 (95,5)	0,125
c) Presence of cognitive impairment	3 (13,6)	9 (40,9)	0,070
d) Palliative care	17 (77,3)	22 (100)	0,063
e) Patient in anasarca	3 (13,6)	-	0,250
f) Terminally ill patient	15 (68,2)	20 (90,9)	0,125
g) Other (dehydration, older adults)	3 (13,6)	1 (4,5)	0,625
**3. Absolute contraindications (admits more than one correct alternative)**			
a) Patient’s refusal	8 (36,4)	21 (95,5)	0,001
b) Anasarca	13 (59,1)	18 (81,8)	0,180
c) Severe thrombocytopenia	4 (18,2)	19 (86,4)	<0,001
d) Need for rapid volume replenishment	9 (40,9)	17 (77,3)	0,021
e) Lesion at the puncture site	13 (59,1)	6 (27,3)	0,065
f) Areas with impaired lymphatic circulation	16 (72,7)	10 (45,5)	0,070
g) Cachexia	5 (22,7)	5 (22,7)	1,000
**4. Relative contraindications (admits more than one correct alternative)**			
a) Anasarca	6 (27,3)	5 (22,7)	1,000
b) Severe pulmonary congestion risk	4 (18,2)	3 (13,6)	1,000
c) Patients with coagulation disorders	7 (31,8)	2 (9,1)	0,180
d) Ascites	6 (27,3)	17 (77,3)	0,003
e) Cachexia	6 (27,3)	19 (86,4)	0,002
f) Presence of cognitive impairment	5 (22,7)	3 (13,6)	0,688
g) Possibility for the patient to stay at home	3 (13,6)	2 (9,1)	1,000
h) Bone prominences	9 (40,9)	20 (90,9)	0,001
i) Joints proximity	12 (54,5)	20 (90,9)	0,008
j) Areas of infection, inflammation, or skin lesion	15 (68,2)	18 (81,8)	0,375
k) Other	-	-	-

* McNemar test (p-value <0.05 - 95% significance)

In relation to the practical aspects of hypodermoclysis, there was also an increase in the number of correct answers after the educational intervention. It is of note that among the correct puncture sites, only the infraclavicular region showed no difference between pre- and post-test (59.1% versus 81.8%; p=0.125). Among the possible complications, local edema (96.4% versus 100%; p 0.250) and local pain/discomfort (54.5% versus 72.7%; p 0.289) also did not differ between evaluations. Regarding the maximum volume to be infused, it was observed that in the initial assessment, many students stated that the maximum was 1000 mL and, after the intervention they realized they were wrong. However, the number of correct answers regarding the infusion of 1500 mL did not differ between groups, as shown in Table 2. 

**Table 3:** - Frequency of student responses about practical aspects of hypodermoclysis before and after the educational intervention. Viçosa, MG, Brazil, 2022

**Practical aspects of hypodermoclysis**	**Pre-test n(%)**	**Post-test n(%)**	p-value*
**1. Puncture sites (admits more than one correct alternative)**			
a) Upper third of the lateral aspect of the arm	14 (63,6)	22 (100)	0,008
b) Gluteal region	2(9,1)	2 (9,1)	1,000
c) Scapular region	10 (45,5)	22 (100)	<0,001
d) Anterolateral region of the thigh	15 (68,2)	21 (95,5)	0,031
e) Abdominal region	15 (68,2)	22 (100)	0,016
f) Infraclavicular region	13 (59,1)	18 (81,8)	0,125
g) Arteries	-	1 (4,5)	1,000
**2. Possible complications (admits more than one correct alternative)**			
a) Local swelling	19 (96,4)	22 (100)	0,250
b) Anasarca	4 (18,2)	1 (4,5)	0,375
c) Local pain/discomfort	19 (86,4)	13 (59,1)	0,070
d) Infection	12 (54,5)	16 (72,7)	0,289
e) Pulmonary congestion	3 (13,6)	1 (4,5)	0,625
f) Hematoma	13 (59,1)	21 (95,5)	0,021
g) Infiltration	12 (54,5)	17 (77,3)	0,267
**3. Responsible for the prescription**			
a) Physician	6 (27,3)	14 (63,6)	0,039
b) Physician or Nurse	10 (45,5)	6 (27,3)	0,289
c) Nurse	1 (4,5)	-	1,000
d) Does not need a specific prescription	-	1 (4,5)	1,000
e) Depends on the institutional protocol	5 (22,7)	1 (4,5)	0,219
**4. Person responsible for the puncture**			
a) Physician	-	1 (4,5)	1,000
b) Nurse	4 (18,2)	-	0,125
c) Physician and nurse	8 (36,4)	2 (9,1)	0,109
d) Physician and nurse and nursing technician	4 (18,2)	13 (59,1)	0,022
e) Any health professional, provided they are qualified	4 (18,2)	6 (27,3)	0,625
f) Depends on the institutional protocol	2 (9,1)	1 (4,5)	1,000
**5. Device used for puncture**			
a) Needle catheter (scalp ^®^ )	4 (18,2)	1 (4,5)	0,375
b) 13x0.45 mm needle	1 (4,5)	-	1,000
c) Non-needle catheter (abocath ^®^ , jelco ^®^ )	2 (9,1)	1 (4,5)	1,000
d) Double lumen catheter	1 (4,5)	-	1,000
e) Needled or non-needled catheter	7 (31,8)	18 (81,6)	0,001
f) Catheter needled, non-needled or 13x0.45 mm needle	7 (31,8)	2 (9,1)	0,125
**6. Dwell time needled device**			
a) From 2 to 3 days	4 (18,2)	2 (9,1)	0,688
b) Up to 5 days	1 (4,5)	18 (81,8)	<0,001
c) Up to 7 days	3 (13,6)	1 (4,5)	0,625
d) Up to 11 days	-	1 (4,5)	1,000
e) After the end of each infusion	10 (45,5)	-	0,002
f) Depends on the medication infused	2(9,1)	-	0,500
g) Cannot be used	2 (9,1)	-	0,500
**7. Dwell time non-needle operated device**			
a) From 2 to 3 days	7 (31,8)	1 (4,5)	0,031
b) Up to 5 days	4 (18,2)	1 (4,5)	0,250
c) Up to 7 days	4 (18,2)	2 (9,1)	0,688
d) Up to 11 days	-	19 (86,4)	<0,001
e) After the end of each infusion	3 (13,6)	-	0,250
f) Depends on the medication infused	4 (18,2)	-	0,125
**8. Maximum volume to be infused in 24 h**			
a) Up to 1000 mL	10 (45,5)	1 (4,5)	0,012
b) Up to 1500 mL	8 (36,4)	13 (59,1)	0,180
c) From 1501 to 3000 mL	1 (4,5)	7 (31,8)	0,070
d) Up to 100 mL	3 (13,6)	1 (4,5)	0,625

* McNemar test (p-value <0.05 - 95% significance)

In the pre and post-intervention groups, the students’ self-assessment of their ability to explain the procedure to another student showed a mean score of 0.9 (± 1.4) and 5.9 (± 2.6) points, respectively. The mean score of the ability to perform the procedure in the pre-intervention group was 1.9 (± 2.9) points, while in the post-intervention group this mean was 5.0 (± 3.4) points. Thus, it was found that the educational intervention using the problematization methodology showed positive and statistically significant results (p<0.001) in the self-assessment of students on the ability to explain and perform hypodermoclysis, which demonstrates security in relation to the knowledge of the technique.

## Discussion

It is known that teaching in higher education institutions has transitioned from a traditional model to the increasing use of active teaching methodologies, so that students become active participants in their knowledge construction ^(^ ^13^ ^)^. This reality corroborates the need for courses in the health area to develop in future professionals relational intelligence, autonomy and responsibility for self-learning ^(^ ^14^ ^)^. 

These skills are increasingly important in a modern healthcare scenario, where collaboration and teamwork are crucial. The active teaching methodologies used impact knowledge acquisition, critical thinking and professional autonomy development ^(^ ^15^ ^)^. 

In this sense, the problematization methodology is seen as a stimulus for the promotion of autonomy among students through the practice of reflection and critical thinking when faced with a reality ^(^ ^16^ ^)^. In the context of hypodermoclysis, the development of reflective thinking becomes even more important, since its applicability occurs, most of the time, in an environment of suffering and difficult decisions, both for the professional and the patient. 

In this study, the problematization’s use based on the Arch of Maguerez aimed to contribute to the acquisition of knowledge, allowing for an interactive and reflective training, thus promoting the development of critical thinking in future health professionals. Based on the analysis of the pre-test, it was found that students had fragile prior knowledge in most of the questions analyzed, especially regarding the contraindications and length of stay of the different devices. A similar result was found in a study conducted with 119 nursing students who assessed their knowledge about the hypodermoclysis technique and found that only 40% of them reported knowing it ^(^ ^17^ ^)^. It is believed that this data can be justified by the fragility of the teaching on hypodermoclysis during professional training ^(^ ^5^ ^)^. 

In addition, health training is marked by the predominance of teaching through traditional expository methodology, which considers the student as a knowledge receiver, not leading him/her to critically reflect on the different contexts in which hypodermoclysis can be used. Changes in the training process are necessary for a change in professional practice. However, among the teachers, there are difficulties in breaking with traditional teaching methodologies, particularly the lack of institutional support for the adoption of new approaches ^(^ ^18^ ^)^. These factors, added to the practical scenario in which professionals do not perform the technique, contribute to the occurrence of a vicious cycle of low adherence to hypodermoclysis. 

When the correct answers obtained in the pre and post-test were compared, a significant improvement was observed in both theoretical and practical aspects. Similarly, a Brazilian study that used problematization as a strategy for teaching indwelling urinary catheterization showed a significant increase in the median number of correct answers after the intervention, which reinforces the positive impact of the methodology used ^(^ ^19^ ^)^. It is believed that the positive impact on the theoretical and practical aspects is due to the students’ involvement in the teaching and learning process foreseen in the problematization methodology. The student is led to identify the problem, study it and autonomously propose a solution. This process of independence generates deepening in the study, contributing to the understanding and apprehension of knowledge. 

It was found that, in this study, the highest number of correct answers permeates theoretical issues such as indications and contraindications, but also practical issues such as puncture sites, professionals responsible for the indication and puncture, devices used and length of stay.

The indication of the technique is one of the fundamental criteria regarding the knowledge to perform hypodermoclysis. It is noteworthy that before the educational activity, few students considered the impossibility of oral ingestion as an indication; however, in the post-test, only one student did not identify this indication, which shows a good performance in the learning of this item after the intervention.

This result has substantial importance for clinical practice, since one of the most common indications for the use of hypodermoclysis is the need for fluid administration in patients with impossibility of oral ingestion. A study with 272 patients identified that impaired oral intake represented 39% of indications for hypodermoclysis ^(^ ^20^ ^)^, convergent with another study conducted with 80 cancer patients in palliative care identified that 47.5% had oral intolerance as an indication for the use of hypodermoclysis ^(^ ^21^ ^)^. This underscores the importance of professional health knowledge about hypodermoclysis as a viable alternative for fluid administration in patients who face difficulties with oral ingestion. 

These patients benefit from technique of hypodermoclysis to subcutaneous access the detriment of other routes, considered more painful and with higher risk of adverse effects, such as the intravenous route. In this sense, another study conducted with 160 cancer patients in palliative care identified that the number of occurrences related to peripheral venipuncture was greater than those related to hypodermoclysis ^(^ ^22^ ^)^. 

In relation to the contraindications, it was found that the problematization contributed significantly to the number of correct answers. However, specific doubts remained about patients with anasarca and with the presence of areas of infection, inflammation or skin lesion. Anasarca is considered an absolute contraindication, because generalized edema compromises medication absorption ^(^ ^5^ ^,^ ^23^ ^)^. On the contrary, in situations of local edema, it is possible to assess another site for puncture. This fact is also observed in the presence of sites of infection, inflammation or injury, and is therefore considered a relative contraindication, because, as in local edema, there are alternative sites where it is possible to perform the hypodermoclysis puncture ^(^ ^23^ ^)^. 

Among the practical issues of hypodermoclysis, it is noteworthy that among the correct puncture sites, only the infraclavicular region showed no difference between pre- and post-test, which can be justified because it is a region with lower volume of subcutaneous tissue, being more suitable for performing medication administration rather than volume infusion ^(^ ^5^ ^)^. 

regarding relation to possible complications, hypodermoclysis proved to be a safe and effective alternative for hydration and medication administration when performed and supervised by an experienced team ^(^ ^1^ ^)^. A cohort study identified that frequency of adverse events may differ at certain timepoints in the illness trajectory among terminal patients, increasing adverse events among those with greater severity ^(^ ^24^ ^)^. In this research local edema and local pain/discomfort were the possible complications that may occur, but they did not differ between assessments. Edema was considered a complication by most of the students already in the application of the pre-test and, as the end of the intervention, all of them recognized this problem. Local pain/discomfort, in turn, was less often mentioned as a complication both in the post-test, which may be related to the fact that this phenomenon is more frequent in other routes of medication administration, such as intravenous ^(^ ^22^ ^)^. 

A randomized clinical trial carried out with 26 patients’, compared the pain experience between subcutaneous and intravenous hydration and identified that the patients’ pain score was significantly lower in the subcutaneous group, being it was less painful than the intravenous route ^(^ ^25^ ^)^. Thus, the fact that this phenomenon is more frequent in other routes may have become a confounding factor for students at the time of the answers. 

It is also noteworthy that after the educational intervention many students realized they were wrong about the maximum volume to be infused; however, the number of correct answers did not differ between groups.

In relation to the self-assessment performed by students, the educational intervention showed positive and statistically significant results (p<0.001), both in the ability to explain and perform hypodermoclysis. This result may be related to the methodology used, which encourages students to seek their own knowledge, making them less dependent on the educator and able to apply problem-based learning to solve everyday problems ^(^ ^26^ ^)^. 

The success of the use of the problematization methodology through the Arch of Maguerez obtained in this study reinforces the contribution of this methodology, which was also observed in other studies on the problematization’s use in health ^(^ ^19^ ^)^. The intervention performed stood out in terms of participatory learning, contributing to the development of discussions about the different aspects involved in the therapeutic use of hypodermoclysis, as well as the students’ knowledge level, considering the theoretical and practical aspects. It becomes necessary that this theme is reinforced in education in order to train professionals who understand the benefits of using hypodermoclysis according to the literature, for the improvement of evidence-based practice. 

Among the limitations of the study, we can consider the low availability of specific studies on the teaching of hypodermoclysis, which hindered the discussion and comparison with similar studies. In addition, the short period of time between the educational intervention and the post-test, not being possible to verify how the analyzed questions would behave in longer periods of interval. The use of a non-probabilistic sample should also be mentioned, which reinforces the importance of interpreting the results with caution, since the number of nursing students is greater than that of medical students.

These findings demonstrate that the teaching-learning process through active methodologies, such as problematization, must be incorporated into educational institutions, with a view to developing critical thinking and taking a leading role in the training of future health professionals. It is believed that its incorporation could provide students with greater scientific knowledge, development of cognitive and attitudinal skills and, consequently, greater adherence to this technique in their clinical practice. Thus, it is expected to contribute to safety, improvement in the quality of life and well-being of patients who need medicines and solutions, normally indicated for intravenous administration, but with their unavailability.

## Conclusion

The results found in this study allow us to infer that the problematization methodology, as a teaching strategy on hypodermoclysis, positively impacted the knowledge of nursing and medical students.

Weaknesses were identified in the students’ prior knowledge regarding the theoretical and practical aspects of hypodermoclysis. After the educational intervention there was significant improvement in knowledge regarding indications, contraindications, puncture sites, responsible for the prescription, responsible for the puncture, devices used and length of stay. These results show that the problematization methodology can be incorporated into the teaching-learning process of nursing and medical students for teaching procedures such as hypodermoclysis.

The teaching-learning process through active methodologies should be encouraged in the health area, aiming to stimulate the development of critical thinking and protagonism in training. The problematization methodology has the potential to increase knowledge and develop cognitive and attitudinal skills in students, thus improving the quality of care.

Further studies are suggested, using the comparison of the problematization methodology with other teaching strategies in order to identify the best interventions and propose improvements in the health training process. In addition, there is a need for studies that seek to investigate whether the knowledge obtained was learned after a longer period of time after the intervention.
